# Atypical case of AL amyloidosis with urinary erythrocyte casts 

**DOI:** 10.5414/CNCS108640

**Published:** 2015-09-23

**Authors:** Orfeas Liangos, Maike Buettner-Herold, Markus Ketteler, Nicolaos E. Madias

**Affiliations:** 1 Klinikum Coburg, III. Medizinische Klinik, Coburg,; 2 Department of Anatomic Pathology, Section on Nephropathology, University of Erlangen Nuremberg, Erlangen, Germany, and; 3 Department of Medicine, Tufts University School of Medicine, and Department of Medicine, Division of Nephrology, St. Elizabeth’s Medical Center, Boston, MA, USA

**Keywords:** urinary sediment, erythrocyte casts, amyloidosis, urinalysis

## Abstract

We present the case of a 73-year-old man who developed an acute, severe febrile illness with multiorgan dysfunction, featuring renal failure, nephrotic-range proteinuria, microhematuria, and a skin rash. Numerous erythrocyte casts were found on urine microscopy. Typically, the finding of urinary erythrocyte casts indicates the presence of an underlying glomerular inflammatory disease. However, on renal biopsy, only amyloid light-chain (AL) amyloidosis and tubular injury were the predominant findings with no signs of glomerular or vascular inflammation. Photomicrographs of urinary sediment as well as renal biopsy histopathology of the presented case are shown. The unusual combination of findings, is then discussed in light of the existing literature on renal amyloidosis as well as erythrocyte casts in conditions other than glomerulonephritis.

## Introduction 

Evaluation of urinary sediment is essential for the diagnostic work-up of many nephrological problems as urinary sediment findings aid in the differential diagnosis of renal disease [1, 2]. Some findings are considered particularly specific for certain groups of diseases, such as the finding of erythrocyte casts, which typically points to the presence of glomerular inflammation or bleeding, as is the case in glomerulonephritis [3, 4, 5, 6]. Here we describe a case of a patient presenting with numerous urinary erythrocyte casts, in whom no glomerulonephritis, but rather renal AL amyloidosis was found on biopsy. This case is then discussed in light of the existing literature on urinary sediment findings in AL amyloidosis and glomerulonephritis. 

## Case report 

A 73-year-old man presented to the Klinikum Coburg in a transfer from another hospital following a fulminant, generalized illness complicated by multiorgan dysfunction. The patient, a native of Germany, felt ill while vacationing in Thailand and was first treated at a local hospital for a 2-day history of nausea, vomiting, double vision, dizziness, and generalized weakness. Past medical history included arterial hypertension, two-vessel coronary artery disease (CAD), a monoclonal gammopathy of undetermined significance (MGUS), and an episode of rhabdomyolysis of unknown cause 9 years prior, complicated by nonoliguric acute renal failure that did not require renal replacement therapy. Because his condition deteriorated, he was transferred to the Bangkok Heart Hospital, Bangkok, Thailand, where he was admitted to an intensive care unit due to progressive multiorgan involvement, including acute kidney injury, upper gastrointestinal hemorrhage, acute non-ST elevation myocardial infarction, hemodynamic instability, and hypoxic respiratory failure. Urinalyses obtained at the time of admission and on subsequent days consistently showed microhematuria and proteinuria. Treatment included intubation and mechanical ventilation, an episode of cardiopulmonary resuscitation and electrical cardiac defibrillation, vasopressor infusions for hemodynamic instability presumed to be due to septic and/or cardiogenic shock, red blood cell transfusions, intermittent hemodialysis as well as several courses of antibiotics and antimycotics (vancomycin, meropenem, fosfomycine, and itraconazole). In addition to copious, purulent respiratory secretions that developed later in the clinical course, a generalized, palpable maculopapular rash was noted on the trunk and extremities and treated with oral colchicine and glucocorticoid-containing topical creams. In the assessment of the treating physicians, sepsis was one of the initial and principal clinical diagnoses, but no focal source of it, other than the lungs, was identified. Following a 3-week clinical course, his condition progressively improved, allowing for discontinuation of circulatory support and extubation; however, hemodialysis was continued until 2 days prior to transfer. 

**Figure 1.**
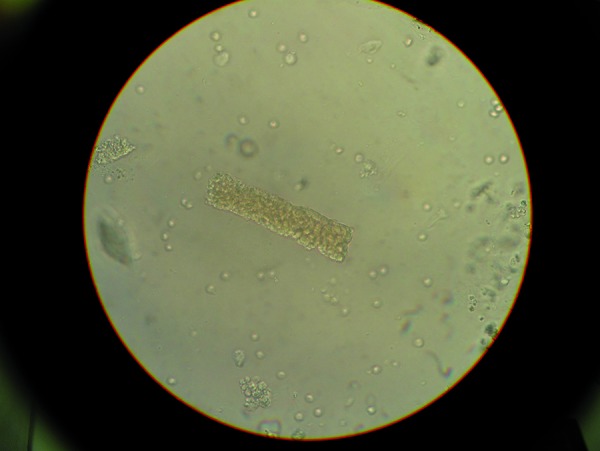
Photomicrograph of a characteristic erythrocyte cast found upon microscopy of the urinary sediment derived from the presented case (40× magnification).

Following repatriation via ambulance flight and upon arrival at the Klinikum Coburg, the patient was awake but disoriented, in no apparent distress, afebrile, hemodynamically stable, and nonoliguric. A 3/6 holosystolic murmur was audible over the base of the heart, without gallops or pericardial rub. Substantially reduced muscle mass and body fat were noted as well as a palpable, nonconfluent, nonpetechial maculopapular rash on both lower extremities below the knees. The remainder of the physical examination was unremarkable. A urinalysis performed immediately upon arrival showed a specific gravity of 1,020 g/L, pH 5, 3+ heme, 4+ protein, trace leukoesterase, 1+ bilirubin, and negative urobilinogen and glucose. Urinary sediment showed multiple, well-formed erythrocyte casts (Figure 1), less numerous granular casts, and a few broad, waxy casts along with 10 – 20 erythrocytes/hpf, 3 – 5 leukocytes/hpf, and no bacteria or crystals. Proteinuria was quantified at 5 g of protein per gram of creatinine. Due to the suspicion of glomerulonephritis, a pulse of corticosteroids was administered and a CT-guided renal biopsy was performed. Renal replacement therapy was continued for solute control. Autoantibody serology tests, including antinuclear, antineutrophil cytoplasmic, and antiglomerular basement membrane antibodies were negative. Serum complement C3 and C4 levels were normal. Serum protein electrophoresis and immunofixations showed an M band identified as IgG λ-light chains. In addition, the serum κ- and λ-light chains were found to be 13 and 130 mg/L, respectively, with a ratio of 0.1 and consistent with the known MGUS. 

### Histological findings 

**Figure 2.**
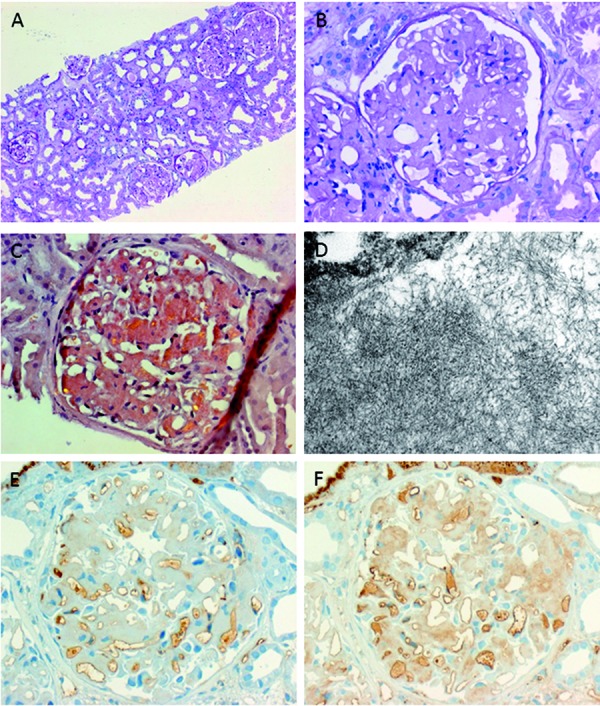
Panel of photomicrographs derived from the presented renal biopsy case. A: overview in periodic acid–Schiff (PAS) stain (100× magnification); B: detail of a glomerulus, PAS stain, (400×); C: detail of a glomerular Congo red stain (400×), note birefringence indicating presence of amyloid deposits; D: electron microscopic preparation showing amyloid fibrils (31,500×). Immunohistochemistry demonstrating presence of λ (E), but not κ (F) light chain deposits (400×); (images provided by Maike Buettner-Herold, M.D., Dept. of Renal Pathology, University of Erlangen, Germany).

The renal biopsy specimen included 29 glomeruli, 8 of which were completely sclerosed. The remainder had diffuse amorphous deposits in the mesangium and the capillary loops that were Congo red-positive with green birefringence under polarized light (Figure 2A, B, C). Such deposits were also found in sections of arterial vessels and faintly in the interstitium. The tubules showed signs of severe acute injury, including dilated lumina, loss of epithelial cell height, and vacuolization. In addition, proximal tubular cells showed signs of protein deposition. There was tubular atrophy in 10 – 15% of the cortical tissue with accompanying interstitial fibrosis. At least 1 definite intratubular erythrocyte cast was found in the biopsy. Only minimal lymphocytic and plasma-cell infiltrates were present in the interstitium. Antibody stains for IgA, IgG, IgM, C1q, and C3c were negative. Electron microscopy confirmed the histopathological diagnosis of renal AL amyloidosis (Figure 2D). There was no evidence of glomerulonephritis or vasculitis. 

### Case follow-up 

The patient gradually regained renal function, renal replacement therapy was discontinued, and his serum creatinine returned to levels of ~ 1.5 mg/dL (eGFR ~ 30 mL/min/1.73 m^2^). Coronary angiography revealed progression of his CAD to three vessels, and a percutaneous transluminal dilatation of the left anterior descending artery with stent placement was performed. In addition, due to clinical and magnetic-resonance-imaging evidence suggestive of amyloid cardiomyopathy as well as the history of ventricular fibrillation, an automated cardioverter-defibrillator was implanted. The patient was discharged with the plan to shortly undergo hematologic follow-up and bone marrow biopsy for work-up and treatment of his plasma-cell dyscrasia. Bone marrow biopsy revealed 10 – 15% infiltration of CD56-positive monoclonal plasma-cell population with lambda-light chain restriction, the remainder of the bone marrow being relatively unaffected with adequate hematopoiesis. The diagnosis of multiple myeloma, type IgG λ, stage III was made [7], and therapy commenced using bortezomib. 

## Discussion 

In this case of severe acute kidney injury requiring renal replacement therapy, gross proteinuria, and microhematuria, the finding of erythrocyte casts upon urinary microscopy points to the presence of glomerulonephritis [2, 3]. Typically, such a diagnosis requires confirmation by renal biopsy before potentially toxic therapies are instituted [8]. 

However, renal histopathology showed renal AL amyloidosis and no signs of glomerular or small-vessel inflammation. This pathologic diagnosis is surprising since renal amyloidosis typically presents as nephrotic syndrome with a bland urinary sediment, except for signs of lipiduria. Rare cases of AL amyloidosis presenting with heavy proteinuria and signs of superimposed tubular injury have been reported [2, 9]. The constellation of findings observed in our case poses two main questions that warrant further discussion: Can renal amyloidosis be associated with glomerular microhematuria? If so, what are the factors associated with such a clinical presentation, and what role could the acute tubular injury play in this context? 

Several cases of renal amyloidosis associated with a nephritic sediment have previously been published. These are virtually exclusively cases of AA amyloidosis that also feature glomerular extracapillary proliferation, a hallmark of crescentic glomerulonephritis [10], mostly in patients with florid rheumatoid arthritis [11, 12, 13, 14, 15, 16]. In these cases, the mechanism of glomerular injury is thought to involve basement membrane rupture and mesangial cell dysfunction due to amyloid-fibril deposition, leading to leakage of proteinaceous material and cellular contents into Bowman’s space, thereby initiating glomerular crescent formation [13]. 

Two other cases of renal AL amyloidosis in association with a nephritic urinary sediment and extracapillary glomerular proliferation on renal biopsy have been reported [17, 18]. In the first report, rapidly progressive deterioration of renal function and glomerular microhematuria developed in a patient with a 4-year history of primary κ-type AL amyloidosis (without lymphoplasmacytoid bone marrow infiltration) and previously biopsy-proven myocardial involvement, who later developed multiple myeloma as evidenced by bone marrow infiltration with 38% plasma cells, a few months prior to developing renal failure. Renal biopsy showed an acute segmental necrosis and cellular crescent formation as well as renal amyloidosis and tubulo-interstitial injury [17]. The second report described a case of AL amyloidosis and Waldenström’s macroglobulinemia in which a nephritic urinary sediment and renal failure developed. Crescentic glomerulonephritis was found at autopsy, showing crescent formation in 80% of the glomeruli with breaks in the glomerular basement membrane colocalizing with amyloid deposits [18]. 

In their textbook, *The Urinary Sediment*, Fogazzi and Verdesca note that microscopic hematuria is “usually absent” in renal amyloidosis, but they do not specify whether they are referring to the AL or the AA type [19]. By contrast, the same group detected microscopic hematuria in ~ 65% of patients with light-chain deposition disease [20]. Nonetheless, Fogazzi and Verdesca concludes that the appearance of a nephritic sediment in both amyloidosis and light-chain deposition disease may herald the superimposition of extracapillary proliferation [19]. 

Our case therefore appears to be the third reported case in the literature where renal AL amyloidosis was associated with a nephritic sediment and renal failure. However, and in contrast to the cases reported by Ogami and Crosthwaite, we found no evidence of glomerular segmental necrosis or cellular crescent formation [17, 18 ]. In contrast, the glomerular pathology in our case was limited to extensive mesangial and glomerular basement membrane amyloid depositions. In addition, our case was associated with λ-light chains, in comparison to κ-light chains in 1 of the previously published cases of AL amyloidosis [17]. Regarding bone marrow pathology, both patients had evidence of pathological infiltration with monoclonal lymphoplasmacytoid cells, whereby infiltration was less pronounced in our case (10 – 15%) in comparison to the case reported previously by Crosthwaite (38%) [17] (key features of these 3 cases are summarized in Table 1). Therefore, and to our knowledge, this is the first reported case of AL amyloidosis associated with multiple myeloma in which acute renal failure with a nephritic sediment was observed without pathologic evidence of extracapillary glomerular proliferation. 

One might suggest that due to the focal manifestation of extracapillary glomerular proliferation, it could have been missed on renal biopsy in our case. Although this possibility cannot be ruled out, the fact that the renal biopsy specimen contained 29 glomeruli renders it unlikely. Because in our case mild changes of interstitial inflammation were found, one could argue that the finding of erythrocyte casts in the urinary sediment could be due to an associated acute interstitial nephritis (AIN). Indeed, erythrocyte casts were found with substantial frequency in a case series of 21 patients with biopsy-confirmed acute interstitial nephritis [5]. The investigators reviewed the urinary sediment findings obtained up to 9 days prior to biopsy in all cases included in that series. Unexpectedly, urinary erythrocyte casts were observed in 6 out of the 21 cases (28.6%). Single case reports have also described the finding of erythrocyte casts in the urinary sediment of patients with biopsy-confirmed AIN [21, 22, 23]. In 1 of these reports, the authors suggest that, particularly in interstitial nephritis involving the distal tubules, the damage to the distal tubular cells and tubular basement membrane may provide the physical pathway from capillary to tubular lumen required for the genesis of erythrocyte casts [23]. In support of this possibility, our patient had been exposed to many drugs, suffered infections, and developed a diffuse rash during his recent illness. However, the typical urinary sediment of acute interstitial nephritis includes large leukocyturia along with erythrocyturia, leukocyte casts, and tubular cells free and in casts, findings that were not present in our case, nor did our patient manifest peripheral blood eosinophilia or eosinophiluria. In addition, the urinalysis obtained upon admission to the Bangkok Heart Hospital already showed microhematuria and proteinuria but no leukocyturia, making an allergic interstitial nephritis, for example in response to the administered antibiotics or antimycotics, unlikely. Furthermore, the interstitial inflammatory changes found in the biopsy specimen were very mild and sparse with only minimal lympho-plasmacellular interstitial infiltrate and without eosinophils or tubulitis. Therefore, the possibility of a coexisting acute interstitial nephritis being the cause of the nephritic sediment observed in our case is extremely improbable. In the absence of a glomerulonephritis, what might be the mechanism of erythrocyte cast formation in our case? One possibility is that red blood cells migrated into the tubular lumens across small arteries, arterioles, and tubular basement membranes affected by the amyloid deposition. Indeed, amyloid deposition was identified along these structures on renal biopsy. 

In summary, we present a case of acute kidney injury, the differential diagnosis of which included a glomerulonephritis due to the finding of erythrocyte casts on urine microscopy. Histologically, and in contrast to our clinical suspicion, acute tubular injury along with underlying renal AL amyloidosis (IgG λ type) was found. Although presence of a nephritic urinary sediment has been described previously in patients with renal amyloidosis, it predominantly involved patients with inflammatory diseases and renal AA amyloidosis. Moreover, almost uniformly, extracapillary glomerular proliferation was a prominent feature on renal biopsy in those cases. Our reported case differs from the previous ones due to presence of renal AL amyloid deposits and absence of glomerular or microvascular inflammation despite the nephritic features upon urinalysis and urine microscopy. 

## Conflict of interest 

The authors have no conflicts of interest pertaining to this work. 

**Table 1.** Comparison of the presented case of renal AL-amyloidosis and nephritic urinary sediment findings with two similar cases published in the literature.

	Current case	Crosthwaite et al. [17]	Ogami et al. [18]
Associated disease	Multiple myeloma	Multiple myeloma	Waldenström’s macroglobulinemia
Urinary sediment	Erythrocyte casts	Erythrocyte casts	Erythrocyte casts
Clinical course	Acute renal failure with recovery	Rapid deterioration of renal function, progression to ESKD	Acute renal failure and death
Renal histology	Glomerular and tubular amyloid deposits, acute tubular injury	Segmental necrosis, cellular crescent formation, renal amyloidosis, tubulointerstitial injury	Crescent formation in 80% of glomeruli, breaks in glomerular basement membranes colocalizing with amyloid deposits

ESKD = end-stage kidney failure.
